# Statin pretreatment diminishes the levels of myocardial ischemia markers not only in CABG

**DOI:** 10.1186/1749-8090-5-131

**Published:** 2010-12-29

**Authors:** José Martínez-Comendador, José Rubio Álvarez, José Benito Garcia Bengochea

**Affiliations:** 1 Department of Cardiovascular Surgery, University Hospital Santiago de Compostela (CHUS). SERGAS. Travesia da Choupana s/n, Santiago de Compostela,15706 A Coruña, Spain

## Abstract

A response to Ege E, Dereli Y, Kurban S, Sarigul A: **Atorvastatin pretreatment diminishes the levels of myocardial ischemia markers early after CABG operation: an observational study**. *J Cardiothorac Surg *2010, **5:**60.

## Correspondence

We read with great interest the manuscript by Ege et al [1] concerning how Atorvastatin pretreatment before CABG diminishes the levels of myocardial ischemia biomarkers.

Our study [2] was the first to report that preoperative treatment with statins reduces biochemical parameters of systemic inflammatory response and myocardial ischemia markers in cardiac surgery with cardiopulmonary bypass (CPB), regardless of being CABG or valvular surgery.

Mannacio et al [3] published the first randomized study showing that pretreatment with rosuvastatin decreases the incidence of myocardial damage in patients undergoing coronary surgery with CPB. In our study Creatine phosphokinase (CPK), CPK-MB and troponin I was assessed at 1, 6, and 24 h after surgery in 138 patients who underwent cardiac surgery with CPB.

The levels observed in the statin treatment group were always lower than those in the group that did not receive treatment, this difference only being significant in the measurement of CPK-MB at 24 h (19.7 ± 23 ng/ml vs 33.1 ± 32.6 ng/ml, p = 0.02) and in the sample collected of Troponin I at the end of the intervention (2.25 ± 2.2 ng/ml vs 3.32 ± 3.1 ng/ml, p = 0.03) and at 24 h (4.15 ± 3.54 ng/ml vs 6.64 ± 8.08 ng/ml, p = 0.04). These findings coincide with the single measurement at 24 h after surgery in the manuscript by Ege et al (for CK-MB levels, 12.9 ± 4.3 versus 18.7 ± 7.4 ng/ml, p = 0.004; for troponin I levels, 1.7 ± 0.3 versus 2.7 ± 0.7 ng/ml, p < 0.001). The higher levels of CPK-MB and Troponin I we found in our study could be explained by the mixed valvular and coronary population, beeing similar with the findings of Landoni G et al [4]. This study demonstrated that each type of cardiac operation has a peculiar amount of myocardial necrosis biomarkers; the highest release of these cardiac biomarkers was associated with mitral valve replacement [4].

Ege et al [1] reports that the study group received minimum 20 mg/kg/day atorvastatin (Ator, Sanovel, Istanbul, Turkey) for at least 15 days before surgery, and we assume that they wish to mean 20 mg/day atorvastatin.

The type of statin we used most was atorvastatin (63.9%) and the most common dose was 20 mg per day at least 3 weeks before surgery [2]. Mannacio et al [3] used 20 mg/day of rosuvastatin one week before surgery. Therefore, it seems possible to achieve the same results, even with low doses of statins and in less time before surgery.

Recent studies performed in patients undergoing cardiac surgery found that statins reduced the mid-term mortality and the number of postoperative complications and clinical events[5]; the common feature of these publications were the large amount of patients necessary to achive clinical results. Ege et al [1] found a shorter duration of ICU stay among patients treated with atorvastatin in an study with only forty cases. In our prospective cohort of 138 patients [2], the different groups analyzed did not show differences with regard to any of the postoperative variables. Therefore, this results should be interpreted cautiously, until future studies with larger sample sizes confirm these findings.

In CABG without myocardial infarction, the amount of cardiac biomarker released seemed to be associated with an increased risk of mortality and late cardiac events[6]. According to this facts an absolute reduction of marker release, as observed in these studies [1-3], could be translated into a reduction of early and late adverse events. The anti-inflammatory action of statins, as we demonstrated [2], its pleiotropic effects and the capacity of reduction of myocardial biomarkers, are the reasons of the potencial beneficial effects of statins in cardiac surgery interventions.

## List of abbreviations

CABG: Coronary artery bypass graft; CPB: Cardiopulmonary bypass; CPK: Creatine phosphokinase.

## Competing interests

The authors declare that they have no competing interests.

## Authors' contributions

The authors read and approved the final manuscript.
